# The Chicken Ovalbumin Upstream Promoter-Transcription Factor II Negatively Regulates the Transactivation of Androgen Receptor in Prostate Cancer Cells

**DOI:** 10.1371/journal.pone.0049026

**Published:** 2012-11-07

**Authors:** Chin-Hee Song, Hyun Joo Lee, Eunsook Park, Keesook Lee

**Affiliations:** Hormone Research Center, School of Biological Sciences and Technology, Chonnam National University, Gwangju, Republic of Korea; University of Saarland Medical School, Germany

## Abstract

Androgen receptor (AR) is involved in the development and progression of prostate cancers. However, the mechanisms by which this occurs remain incompletely understood. In previous reports, chicken ovalbumin upstream promoter-transcription factor II (COUP-TF II) has been suggested to play a role in the development of cancers. In the present study, we explored a putative role of COUP-TF II in prostate cancers by investigating its effect on cell proliferation and a cross-talk between COUP-TF II and AR. Overexpression of COUP-TF II results in the inhibition of androgen-dependent proliferation of prostate cancer cells. Further studies show that COUP-TF II functions as a corepressor of AR. It represses AR transactivation on target promoters containing the androgen response element (ARE) in a dose-dependent manner. In addition, COUP-TF II interacts physically with AR *in vitro* and *in vivo.* It binds to both the DNA binding domain (DBD) and the ligand-binding domain (LBD) of AR and disrupts the N/C terminal interaction of AR. Furthermore, COUP-TF II competes with coactivators such as ARA70, SRC-1, and GRIP1 to modulate AR transactivation as well as inhibiting the recruitment of AR to its ARE-containing target promoter. Taken together, our findings suggest that COUP-TF II is a novel corepressor of AR, and provide an insight into the role of COUP-TF II in prostate cancers.

## Introduction

The normal growth, differentiation, and function of the prostate gland are largely regulated by androgens, which act through androgen receptor (AR) [1], [2]. The inhibition of AR activity by any means, including castration and anti-androgen treatment, can impede or abolish all phases of prostate development [3]. AR function can be modulated by intracellular signaling pathways, transcription factors, cell cycle proteins, and other factors, which modify AR transcriptional activity or provide means for cross-talk between androgen and other signals [4]. Androgens and AR also play an integral role in the growth of prostate tumors [5], [6]. The progression of prostate cancer occurs via the alternation of the normal androgen axis by the dysregulation of AR activity through signal transduction cascades, alterations in AR coregulator expression, and mutations in AR [7].

AR, a ligand-dependent transcription factor, regulates the expression of target genes when activated by androgens [1]. AR consists of three separate functional domains: the N-terminal activating domain, the middle DNA-binding domain, and the C-terminal ligand binding domain [8]. The N-terminus has been shown to directly interact with the C-terminus in a ligand-dependent manner, which is required for the full transcriptional potential of AR [9]. Prior to androgen exposure, AR binds to a multi-protein chaperone complex in its inactive state. Androgen binding induces a conformational change in the AR which results in dissociation from the chaperone complex, dimerization, and translocation into the nucleus, thereby binding to AREs in the regulatory regions of target genes [9]–[11]. AR transcriptional activity is modulated by coregulatory proteins. The ARE-bound AR homodimer recruits coactivators, such as p160 and p300/CBP, which bridge interactions with the general transcription machinery and modify histones, thus effecting the activation of gene expression [12]–[16]. In contrast, corepressors may recruit histone deacetylase (HDAC) to the AR complex, thereby maintaining the chromatin structure [17], [18]. They may also inhibit the functional interaction of the general transcription factors with the promoter [16].

The chicken ovalbumin upstream promoter-transcription factors (COUP-TFs) are orphan members of nuclear receptor superfamily that activate or repress gene transcription by directly binding DNA sequence [19]. There are three members of the human COUP-TF family: COUP-TF I (NR2F1), COUP-TF II (NR2F2), and ErbA-related protein 2 (NR2F6) [20]. COUP-TF I and COUP-TF II proteins are 95% homologous and evolutionarily conserved in the DNA binding domain as well as the ligand-binding domain, mainly differing at the N-terminus (reviewed in [19]). COUP-TF I is more highly expressed in neuronal tissues of the central and peripheral nervous systems, whereas the COUP-TF II is more highly expressed in developing organs such as the lung, kidney, pancreas and prostate [20], [21]. ErbA-related protein 2 is less conserved and little is known about its expression and function [22].

COUP-TF interacts with other nuclear receptors, including estrogen receptor (ER), the retinoid X receptor (RXR), peroxisome proliferator-activated receptors (PPAR), and the vitamin D receptor (VDR) [23]–[27]. In general, COUP-TF inhibits the transcriptional activity of other nuclear receptors by competing for their DNA binding sites or by heterodimerization with the class II nuclear receptor heterodimer partner retinoid X receptor, thereby preventing gene expression [28]. In addition, like thyroid hormone receptor and retinoic acid receptor, unliganded DNA-bound COUP-TF I represses gene expression by an active silencing domain within the ligand-binding domain that recruits corepressors (i. e., NCoR and SMRT), a process called active repression [29]. Finally, COUP-TF can also repress transcription by directly binding to the ligand-binding domain of nuclear hormone receptors (transrepression; [30], [31]). In previous reports, COUP-TF was related with the development of various cancer including breast cancer [24], [32]–[34], lung cancer, and adrenal cancer progression [35]–[37].

In the present study, we demonstrate that COUP-TF II represses the transactivation of AR in prostate cancer cells, resulting in the inhibition of androgen-dependent cell growth. COUP-TF II directly binds AR, preventing the N/C terminal interaction of AR. Furthermore, COUP-TF II inhibits the ligand-induced recruitment of AR to the PSA promoter and competes with AR coactivators to modulate AR transactivation. All together, our results suggest COUP-TF II as a potent AR corepressor and provide an insight into the role of COUP-TF II in prostate cancers.

## Materials and Methods

### Reagents

Antibodies for AR (sc-815), PSA (sc-7638), HA (sc-7392), GFP (sc-9996), and α-tubulin (sc-5286) were obtained from Santa Cruz Biotechnology, Inc. and antibody for AR (PG-21, 06-680) was obtained from EMD Millipore Corporation. Antibody for COUP-TF II (PP-H7147-00), which does not recognize COUP-TF I [19], was purchased from Perseus Proteomics Inc. Radiolabeled thymidine ([methyl-^3^H]-thymidine, specific activity 80 Ci/mmol) was obtained from Perkin Elmer Life Science. Trichostation A was purchased from Sigma-Aldrich Co., and Sodium butylate and Nicotinamide (NIC) were purchased from Calbiochem.

### Plasmids and Construction

The mammalian expression plasmids of mouse AR (pcDNA3-AR), pcDNA3-ARA70, pcDNA3-p300, pSG5HA GRIP1, and pCR3.1 SRC-1, and MMTV-Luc and PSA-Luc reporter plasmids have been previously described [38]. The pPBARE×7-tk-Luc was constructed by inserting the eight copies of androgen receptor response element (ARE) of mouse probasin (PB) gene. HA-tagged mouse COUP-TF II was constructed by insertion of *Msl*I/*Xho*I-digested fragment from pCR3.1-mouse COUP-TF II into *Eco*RV/*Xho*I-digested HA epitope-tagged pcDNA3 vector (pcDNA3HA). GFP-COUP-TF II and GST-COUP-TF II full length were subcloned by insertion of *Eco*RI/*Xho*I-digested fragment from pcDNA3HA-COUP-TF II into *Xma*I-digested pEGFP-C1 vector and *Eco*RI/*Xho*I-digested pGEX4T-1 vector, respectively. GST-COUP-TF II full length and deletion mutants, AF1, DBD+hinge (DBDh), and ΔAF1 regions, were constructed by self-ligation of *Sma*I/*Xho*I-, *Sph*I/*Xho*I-, and *Eco*RI/*Sma*I-digested fragment from GST-COUP-TF II full length, respectively. The mammalian expression plasmids VP-AR1-660 and GAL-AR624-919 and the reporter construct 5XGAL4-Luc3 (originally from Dr. Donald McDonnell) were kindly provided as gifts by Dr. Elizabeth M. Wilson (University of North Carolina) [39].

### Preparation of Recombinant Adenovirus

For the ectopic expression of the mouse COUP-TF II, an adenoviral delivery system was used [40]. Briefly, the COUP-TF II cDNA was cloned into pAdTrack-CMV shuttle vector. Homologous recombination was performed by transformation of adEasy-BJ5138 competent cells with pAdTrack-CMV-COUP-TF II together with adenoviral gene carrier vector. The recombinant viruses were selected, amplified in HEK 293 cells, and purified by cesium chloride density centrifugation. Viral titers were measured using Adeno-X rapid titer (BD Biosciences) according to the manufacturer’s instructions.

### Cell Culture and Transient Transfection Assay

COS-7 and PPC-1 cells were maintained in Dulbecco's minimum essential medium (DMEM) (Life Technologies, Inc.) supplemented with 10% FBS and 100 units/ml penicillin/streptomycin. LNCaP cells (American Type Culture Collection) were maintained in RPMI 1640 medium (Life Technologies Inc.) supplemented with 10% FBS, 100 units/ml of penicillin/streptomycin, and 2 mM L-glutamine.

Twenty-four hours prior to transfection, cells were plated in 24-well plates and transfected with the indicated amount of expression plasmids, a reporter construct and the control lacZ expression plasmid pCMVß using the SuperFect (Qiagen) or Lipo2000 (Invitrogen) transfection reagent. Total amounts of expression vectors were kept constant by adding appropriate amounts of the depleted vector. Twenty-four hours after transfection, the medium was replaced with fresh medium containing 10% charcoal-stripped serum and either DHT or vehicle. Cells were harvested 24 h after the addition of hormone, and luciferase and ß-galactosidase activities were assayed as previously described [41]. The levels of luciferase activity were normalized to the lacZ expression.

### RT-PCR and qRT-PCR

Total RNA was extracted from the prostate with Tri reagent solution (Molecular Research Center, Inc.). For RT-PCR, 1 µg of total RNA was reverse-transcribed and PCR-amplified with COUP-TF II-specific primers, which amplify a 650 bp fragment spanning ORF. Quantitative analysis of PSA gene expression in LNCaP cells infected with AdGFP or AdCOUP-TF II was assessed by qRT-PCR with PSA-specific primers, which amplify a 517 bp region spanning ORF, using a SYBR Green PCR kit and a Rotor-Gene RG3000 Real-Time PCR system (Corbett Research). As an internal control, PCR reactions were also performed using β-actin-specific primers, which amplify a 362 bp region spanning exon 4. The oligonucleotide sequences were as follows: forward 5′-AAGCTGTACAGAGAGGCAGGA-3′ and reverse 5′-AGAGCTTTCCGAACCGTGTT-3′ for COUP-TF II; forward 5′-GGCCAGGTATTTCAGGTCAG-3′ and reverse 5′-CCACGATGGTGTCCTTGATC-3′ for PSA; and forward 5′- GAGACCTTCAACACCCCAGCC -3′ and reverse 5′- CCGTCAGGCAGCTCATAGCTC -3′ for β-actin.

### GST Pull-down Assay

GST, GST-AR domain mutants, and GST-COUP-TF II deletion mutants were expressed in *E. coli* BL21 cells and isolated with glutathione-Sepharose-4B beads (Pharmacia, Biotech AB). Immobilized GST fusion proteins were then incubated with [^35^S] methionine-labeled COUP-TF II or AR proteins produced by *in vitro* translation using the TNT-coupled transcription-translation system (Promega). The binding reactions were carried out in 250 µl of GST-binding buffer (20 mM Tris-HCl at pH 7.9, 250 mM NaCl, 10% glycerol, 0.05% NP-40, 5 mM MgCl_2_, 0.5 mM EDTA, 1 mM DTT, and 1.5% BSA) overnight at 4°C. The beads were washed five times with 1 ml of GST-binding buffer. Bound proteins were eluted by the addition of 20 µl of SDS-PAGE sample buffer, and were analyzed by SDS-PAGE and autoradiography [41].

In order to determine interfering with the interaction occurring between DBD and LBD of AR by COUP-TF II, we conducted GST pull-down competition assay. Immobilized GST-AR LBD proteins were incubated with [^35^S] methionine-labeled AR AF1DBDh proteins produced by *in vitro* translation. For competition analysis, 2, 5, and 10-fold excess of *in vitro* translated COUP-TF II proteins was added together with radiolabeled AR AF1DBDh proteins.

### Coimmunoprecipitation and Western Blot Analysis

*In vivo* coimmunoprecipitation assay was performed with PPC-1 cells transfected with 5 µg of AR and 5 µg of GFP-COUP-TF II expression plasmids. Transfected cells were treated with 10 nM DHT or vehicle for 4 h post-transfection and harvested with RIPA cell lysis buffer (50 mM Tris-HCl at pH 8.0, 150 mM NaCl, 1% NP-40, 1 µg/ml aprotinin, 0.1 µg/ml leupeptin, 1 µg/ml pepstatin, 0.1 mM PMSF). Whole cell lysate (800 µg) was incubated with 20 µl of protein A/G plus agarose bead slurry (Santacruz) to exclude nonspecific binding and was then centrifuged. The supernatant was divided into two equal portions. One portion was incubated with 2 µg of anti-AR antibody (sc-815) and the other was incubated with 2 µg anti-GFP antibody (sc-9996) overnight at 4°C. Each portion was further incubated for another 4 h after the addition of 20 µl of protein A/G plus agarose bead slurry (Santacruz). Agarose beads were washed four times each with RIPA buffer at 4°C, and bound proteins were separated by SDS-PAGE. Proteins on the gels were transferred to Protran nitrocellulose transfer membrane (Schleicher and Schuell Bioscience), and subjected to Western blot analysis with anti-AR (sc-815) and anti-GFP (sc-9996) antibodies. Signals were then detected with an ECL kit (Amersham Pharmacia).

### Chromatin Immunoprecipitation (ChIP) Assay

LNCaP cells grown in RPMI 1640 medium containing 10% charcoal-stripped serum were infected with either AdCOUP-TF II or AdGFP, and the cells were treated with 10 nM DHT or vehicle for 6 h. Cells were than cross-linked with 1% formaldehyde, and processed for ChIP assay as previously described [42]. Anti-AR antibody (PG-21) was used for immunoprecipitation. Immunoprecipitated DNA and input-sheared DNA were subjected to PCR using a specific primer pair (forward: 5′-CATGTTCACATTAGTACACCTTGCC-3′ and reverse: 5′-TCTCAGATCCAGGC TTGCTTACTGTC-3′), which amplifies a 315 bp region spanning the AR binding site of the PSA enhancer region [43]. As a negative control, PCR reactions were performed using an actin primer pair (forward: 5′-GAGACCTTCAACACCCCAGCC-3′ and reverse: 5′-CCGTCAGGCA GCTCATAGCTC-3′), which amplifies a 362 bp region spanning exon 4 of the β-actin gene.

### Immunofluorescence

The day before transfection, PPC-1 was plated onto gelatin-coated coverslips. RFP-tagged AR and GFP-tagged COUP-TF II was transiently transfected using SuperFect reagent (Qiagen). After 4 h, fresh medium was added to the cells. Twenty-four hours after transfection, the cells were fed with fresh medium with 10 nM DHT or vehicle and incubated for another 4 h. Cells were then washed three times with cold PBS and fixed with 2% paraformaldehyde for 10 min. Fixed cells were mounted on glass slides and observed under laser scanning confocal microscope (LSM 5 PASCAL Laser Scanning Microscope, Zeiss).

### Thymidine Incorporation

LNCaP cells were cultured in 24-well plates at a density of 2×10^4^ cells per well and infected with either AdCOUP-TF II or AdGFP in 10% charcoal-stripped serum-supplied medium. After sitting overnight, the cells were treated with 10 nM DHT for 24 h and then pulse labeled with [^3^H]-thymidine (10 µCi/ml, specific activity 80 Ci/mmol, PerkinElmer Life Sciences, Norwalk, CT) for 4 h. Cells were harvested onto a glass microfiber filter (Whatman, Inc., Florham Park, NJ) and intensively washed with distilled water. Incorporation of thymidine into DNA is measured by counting the filters with a scintillation counter.

### Soft Agar Colony Formation

LNCaP cells were infected with either AdCOUP-TF II or AdGFP in 10% charcoal-stripped serum-supplied medium. After 24 h of infection, the cells were trypsinized and seeded at 5×10^3^ cells in 0.35% agar over 0.7% agar layer in six-well culture dishes. Fresh complete growth medium or charcoal-stripped serum medium containing absence or present of 1 nM DHT was changed every 2 days for 2 weeks. Colonies larger than diameter of 300 mm were scored.

### Statistical Analysis

A statistical analysis was performed by utilizing Student’s t-test with the PRISM software system for Windows. In all cases probability (P) values below 0.05 were considered significant.

## Results

### COUP-TF II Overexpression Represses the Proliferation of Prostate Cancer Cells

**Figure 1:**
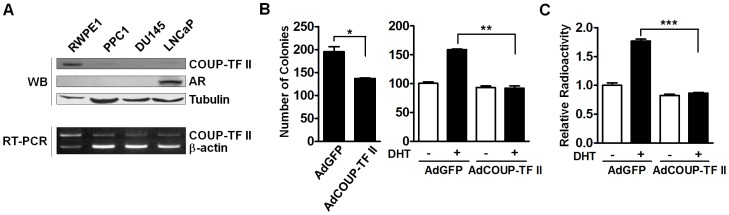
COUP-TF II inhibits androgen-dependent proliferation of prostate cancer cells. (A) COUP-TF II expression in human prostate cancer cell lines. Protein expression levels of COUP-TF II were determined by Western blot analysis of total proteins using anti-COUP-TF II, anti-AR (sc-815), and anti-α-tubulin antibodies. mRNA expression levels of COUP-TF II were determined by RT-PCR of total RNAs. The expression of tubulin and β-actin was used as an internal control. (B) COUP-TF II inhibits the growth of prostate cancer cells. Soft agar colony formation assay was conducted with complete growth medium (left panel) or with medium containing charcoal-stripped serum and supplemented with or without 1 nM DHT (right panel). LNCaP cells were infected with AdGFP or AdCOUP-TF II for 24 h, and were processed for colony formation assay as indicated in “Materials and Methods”. Colonies larger than diameter of 300 mm were scored. Data are representative of three independent experiments. (C) COUP-TF II decreases the rate of DNA synthesis in prostate cancer cells. LNCaP cells were infected with AdGFP or AdCOUP-TF II in medium containing charcoal-stripped serum and supplemented with or without 1 nM DHT. Their DNA synthesis rate was then analyzed by [^3^H]-thymidine incorporation assay. At least three independent experiments were combined and values represent the mean±SEM. *, P<0.05; **, P<0.01; ***, P<0.001.

COUP-TF II is highly expressed in the mesenchymal compartments of developing organs including the prostate [20], [21]. In addition, COUP-TF II has been suggested to play a role in the development of cancer [24], [32], [33], [35]–[37]. Therefore, we initially investigated the expression of COUP-TFs in prostate cancer cell lines and also a role in the proliferation of prostate cancer cells. COUP-TF II was highly expressed in a normal prostate cell line, RWPE1, but its expression was hardly detectable or very low in prostate cancer cell lines, both androgen-dependent and androgen-independent (Figure 1A).

Because COUP-TF II was expressed at very low level in prostate cancer cell lines, we postulated that COUP-TF II might inhibit the proliferation of prostate cancer cells. To test this hypothesis, we infected androgen-dependent LNCaP cells with AdGFP or AdCOUP-TF II, and checked cell proliferation rate by soft agar colony formation assay. Overexpression of COUP-TF II significantly decreased the colony number as well as colony size of LNCaP cells in complete growth medium (Figure 1B, left panel). We then investigated whether COUP-TF II affects the androgen-dependent proliferation of LNCaP cells. Overexpression of COUP-TF II completely inhibited DHT-dependent colony formation of LNCaP cells in medium containing charcoal-stripped serum (Figure 1B, right panel). Inhibition of androgen-dependent growth by COUP-TF II was further confirmed in [^3^H]-thymidine incorporation assay. COUP-TF II expression completely blocked androgen-induced DNA synthesis in LNCaP cells (Figure 1C). These results suggest that COUP-TF II inhibits the androgen-dependent growth of prostate cancer cells.

### COUP-TF II Represses AR Transactivation

**Figure 2:**
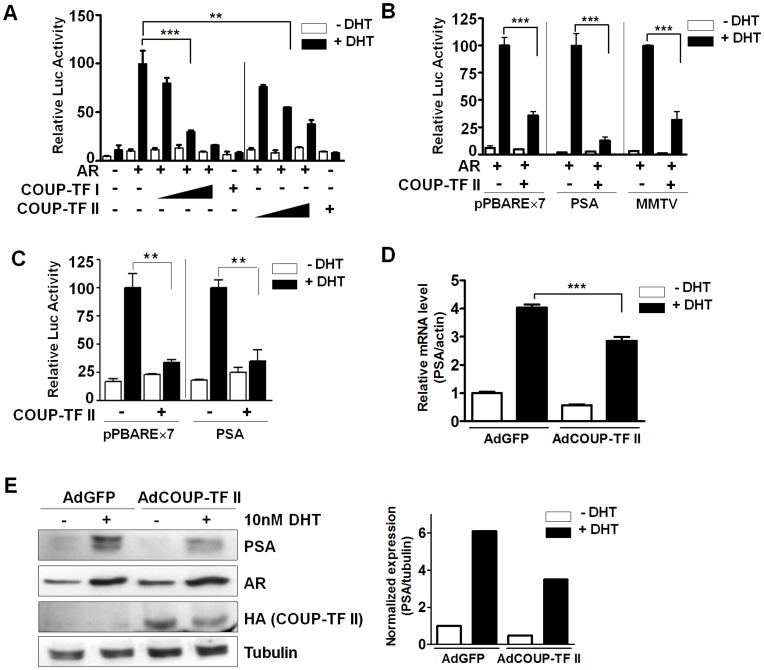
COUP-TF II represses the transactivation of AR. (A) Dose-dependent inhibition of AR transactivation by COUP-TFs. PPC-1 cells were cotransfected with 350 ng of pPBARE×7-tk-Luc reporter and 50 ng of AR expression plasmid along with increasing concentration (100, 250, and 500 ng) of COUP-TF I or COUP-TF II. Cells were treated with or without 3 nM DHT for 24 h. (B) COUP-TF II-mediated repression of AR transactivation on natural AR-target promoters. PPC-1 cells were transfected as in “A”, with PSA-luc or MMTV-luc reporter. (C) COUP-TF II-mediated repression of endogenous AR transactivation. LNCaP cells were transfected as in “A”, without the AR expression plasmid. (D) Repression of androgen-induced PSA mRNA expression by COUP-TF II. LNCaP cells were infected with AdGFP or AdCOUP-TF II. After 24 h of recovery, the cells were treated with 10 nM DHT, and cultured for another 24 h prior to harvesting. Quantitative RT-PCR analysis was conducted using specific primers for PSA and β-actin. The relative PSA mRNA expression was normalized by β-actin expression. At least three independent experiments were combined and values represent the mean±SEM (A–D). **, P<0.01; ***, P<0.001. (E) Repression of androgen-induced PSA protein expression by COUP-TF II. LNCaP cells were infected and processed as in “D”, and cultured for another 48 h prior to harvesting. Western blot analysis of total proteins was conducted using anti-PSA, anti-AR (sc-815), anti-HA, and anti-α-tubulin. Data are representative of three independent experiments (left). The relative PSA protein expression was quantified by normalizing with tubulin expression (rignt).

Since the COUP-TF II overexpression inhibits the androgen-dependent growth of LNCaP prostate cancer cells, we investigated a possible cross-talk between COUP-TF II and AR, which is important for the development of prostate cancers. To test for a cross-talk, we coexpressed COUP-TF II with AR in the PPC-1 cell line, a PC-3 derivative and AR-negative [44], and accessed the effect on the transactivation potential of AR. As shown in Figure 2A, COUP-TF II inhibited androgen-dependent AR transactivation in a dose-dependent manner. COUP-TF I also strongly inhibited AR transactivation, but it was expressed neither in mouse prostate (data not shown) nor in prostate cancer cell lines [20], [21].

In order to establish the importance of COUP-TF II-mediated AR repression, we examined COUP-TF II effect on natural AR-target promoters such as MMTV and PSA. In PPC-1 cells, coexpression of COUP-TF II with AR repressed AR transactivation on both MMTV and PSA promoters (Figure 2B). Furthermore, COUP-TF II also represses the endogenous AR transactivation on minimal ARE promoter AREx7 and PSA promoter in LNCaP cells that express the mutated, but functional, AR (Figure 2C).

PSA is the best characterized androgen-responsive gene as well as a prostate-specific tumor marker. Thus, we assessed the effect of COUP-TF II on the expression of endogenous PSA in AR-positive LNCaP cells, which were infected with COUP-TF II expressing adenovirus (AdCOUP-TF II). Overexpressed COUP-TF II significantly downregulated the androgen-induced expression of endogenous PSA mRNA (Figure 2D) and protein (Figure 2E), while it had no effect on AR protein expression. Together, these results indicate that COUP-TF II represses AR function in prostate cancer cells, inhibiting the expression of endogenous AR target gene PSA.

### COUP-TF II Physically Interacts with AR *in vitro* and *in vivo*

**Figure 3:**
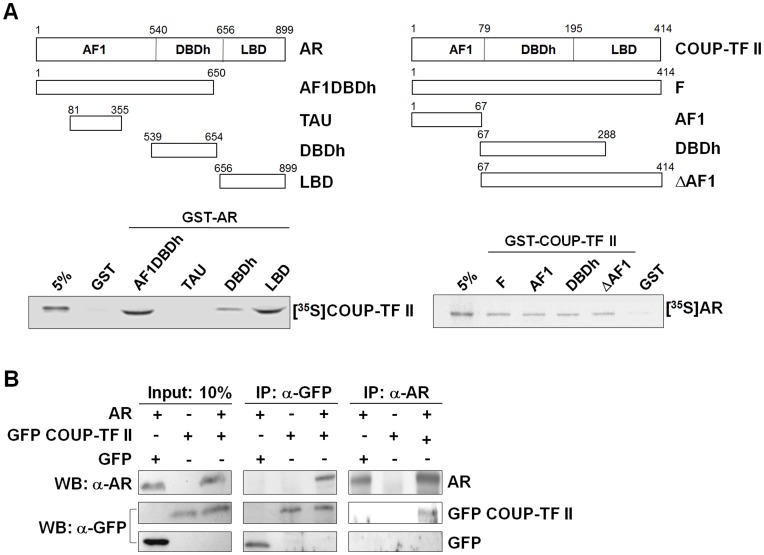
COUP-TF II physically interacts with AR *in vitro* and *in vivo*. (A) Direct interaction between COUP-TF II and AR. Left upper panel, Schematic representation of the full-length AR and its different domain deletion mutants used in GST pull-down assay. Left lower panel, COUP-TF II directly interacts with AR via the DBDh, and LBD region of AR. [^35^S] methionine-labeled COUP-TF II was allowed to bind with bacterially expressed GST alone or with different domain deletion mutants of AR (GST-AR AF1DBDh, GST-AR TAU, GST-AR DBDh, GST-AR LBD). Reactions were carried out with the equivalent amount of each protein as determined by Coomassie blue staining (data not shown). Five percent of the labeled protein used in the binding reaction was loaded as input. Right upper panel, Schematic representation of full-length COUP-TF II and its deletion mutants. Right lower panel, AR directly interacts with COUP-TF II. [^35^S] methionine-labeled AR was allowed to bind with GST alone, the full length (GST-COUP-TF II F) or different deletion mutants of COUP-TF II (GST-COUP-TF II AF1, GST-COUP-TF II DBDh, and GST-COUP-TF II ΔAF1). Data are representative of three independent experiments. F: Full length of COUP-TF II; AF1DBDh: AF1+DBD+hinge; TAU: transactivation unit; DBDh: DBD+hinge region. (B) COUP-TF II is coimmunoprecipitated with AR. PPC-1 cells were transfected with AR and GFP-fused COUP-TF II expression plasmids and then treated with or without 10 nM DHT for 24 h post-transfection. Coimmunoprecipitations were conducted with anti-AR (sc-815) or anti-GFP antibody. Western blot analyses of immunoprecipitated materials were performed using anti-AR (sc-815) or anti-GFP antibodies. Input blots are shown for the expression level of each protein. Data are representative of three independent experiments.

GST pull-down assay was performed in order to examine whether AR repression by COUP-TF II is mediated through direct protein-protein interaction. Interactions of AR with COUP-TF II, as well as AR domains responsible for the interaction, were investigated using different AR deletion mutants fused to the GST protein (Figure 3A, left panel). The *in vitro* translated COUP-TF II interacted with GST-AR AF1DBDh, GST-AR DBDh, and GST-AR LBD, but not with GST-AR TAU, suggesting the involvement of the DBDh and LBD domains of AR in its interaction with COUP-TF II. COUP-TF II domains responsible for its interaction with AR were then investigated using GST fusion protein of COUP-TF II deletion mutants (Figure 3A, right panel). The *in vitro* translated AR interacted with the full-length COUP-TF II and the deletion mutants (COUP-TF II AF1, COUP-TF II DBDh and COUP-TF II ΔAF1), suggesting AR interaction with multiple domains of COUP-TF II.

To examine *in vivo* interaction between COUP-TF II and AR, we performed coimmunoprecipitation assay with PPC-1 cells which were cotransfected with AR and GFP-fused COUP-TF II expression plasmids. Immunoprecipitations using anti-AR or anti-GFP antibody, followed by Western blot analysis of the immunoprecipitated complexes for AR and COUP-TF II, revealed that AR and COUP-TF II were efficiently coprecipitated (Figure 3B).

### COUP-TF II Interferes with the N/C-terminal Interaction of AR

**Figure 4:**
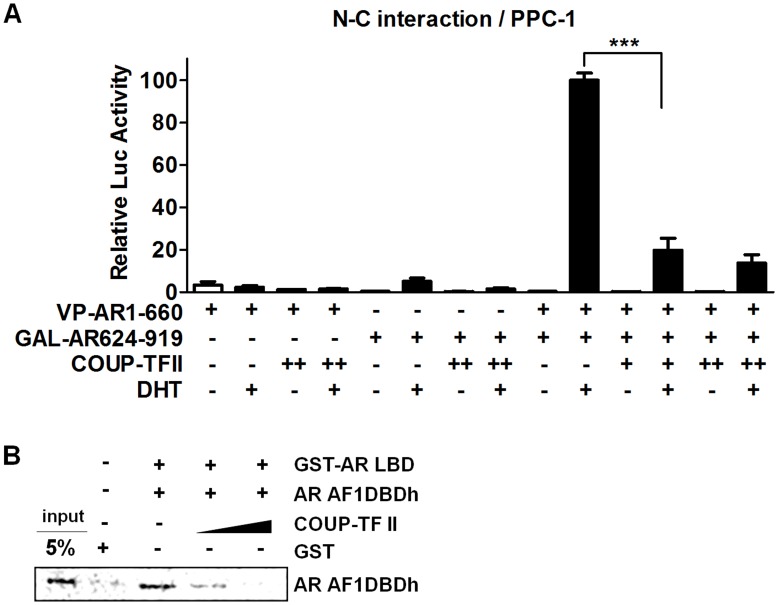
COUP-TF II inhibits the N/C terminal interaction of AR. (A) Mammalian two-hybrid assay. PPC-1 cells were transfected with 5XGAL4-Luc3 together with or without VP-AR1-660, GAL-AR624-919, and COUP-TF II expression plasmids. Cells were treated with or without 10 nM DHT for 24 h. At least three independent experiments were combined and values represent the mean±SEM. ***, P<0.001. (B) GST pull-down competition assay. Immobilized GST-AR LBD proteins were incubated with [^35^S] methionine-labeled AR AF1DBDh proteins produced by *in vitro* translation. For competition analysis, 5 and 10-fold excess of *in vitro* translated COUP-TF II proteins was added together with radiolabeled AR AF1DBDh proteins. Data are representative of three independent experiments. AF1DBDh: AF1+DBD+hinge region.

Upon ligand binding, AR dissociates from heat shock proteins and translocates into the nucleus, thereby binding to its target gene promoters as a homodimer which is formed by the intermolecular N/C terminal interaction of two AR molecules. Because some AR corepressors interfere with the steps involved in androgen-dependent AR activation consequently repressing AR transactivation potential [45], the ability of COUP-TF II to inhibit any of the AR activation steps, such as the N/C terminal interaction and the nuclear translocation of AR, was investigated. PPC-1 cells were transfected with VP-AR1-660 (containing AR residues 1–660), GAL-AR624-919 (containing AR residues 624–919), and increasing amounts of COUP-TF II expression plasmid, along with a luciferase reporter gene regulated by tandem Gal4-responsive elements (5XGAL4-Luc3) [39]. As shown in Figure 4A, COUP-TF II inhibits the interaction of Gal4-AR624-919 with VP-AR1-660 in mammalian two-hybrid system. These results suggest that COUP-TF II inhibits the dimerization of AR through the N/C binding. In order to determine whether COUP-TF II interferes physically with the interaction occurring between N-terminus and C-terminus of AR, we conducted GST pull-down competition assay using GST-AR LBD, *in vitro* translated [^35^S]methionine-labeled AR AF1DBDh, and *in vitro* translated COUP-TF II proteins. AR AF1DBDh was shown to interact with GST-AR LBD, and the interaction was interfered with by COUP-TF II in a dose-dependent manner (Figure 4B).

### COUP-TF II-induced AR Repression was not Related with Nuclear Translocation of AR and HDAC Recruitment

**Figure 5:**
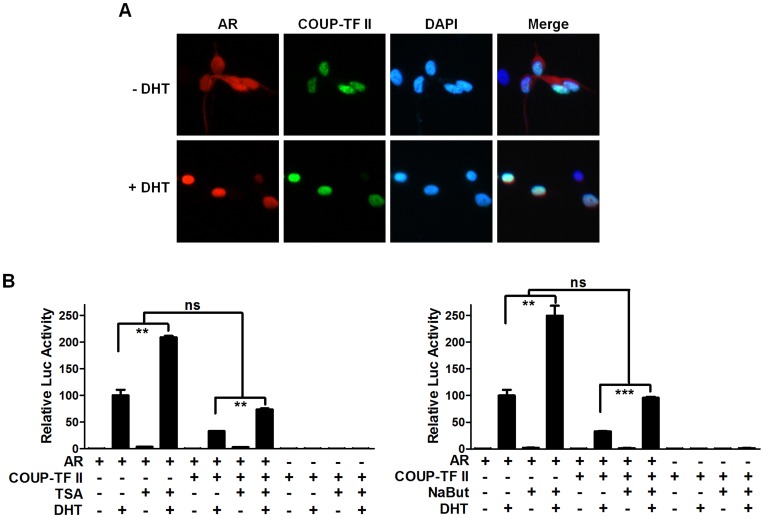
COUP-TF II-induced AR repression involves neither the mislocalization of AR nor recruitment of HDACs. (A) Effects of COUP-TF II on the subcellular localization of AR. PPC-1 cells were transfected with RFP-fused AR and GFP-fused COUP-TF II expression plasmids. Twenty-four hours after transfection, the cells were treated with 10 nM DHT or vehicle for 4 h. Fluorescence was analyzed with a laser scanning confocal microscope. The cellular nuclei were stained with DAPI (blue). Data are representative of three independent experiments. (B) HDAC activity is not involved in the COUP-TF II-mediated repression of AR transactivation. PPC-1 cells were transfected as in Figure 2A. The cells were treated with or without 100 nM TSA or 5 mM NaBut in the presence or absence of 10 nM DHT 24 h prior to harvesting. At least three independent experiments were combined and values represent the mean±SEM. ns, not significant; **, P<0.01; ***, P<0.001.

The effect of COUP-TF II on AR nuclear translocation was assessed by coexpressing RFP-tagged AR and GFP-tagged COUP-TF II in COS-7 cells. When RFP-AR and GFP-COUP-TF II were coexpressed, AR protein was predominantly located in the cytoplasm in the absence of ligand, but, AR protein translocated into the nucleus in the presence of 10 nM DHT (Figure 5A). Irrespective of DHT, COUP-TF II was predictably located in the nucleus. Therefore, neither AR nor COUP-TF II protein was mislocalized by their coexpression. These results suggest that AR repression by COUP-TF II is not likely due to the nuclear exclusion of AR.

Corepressors of nuclear receptors are now known to utilize multiple mechanisms to repress the transactivation of nuclear receptors. They include the recruitment of histone deacetylase (HDAC), which also targets non-histone proteins including transcription factors and coregulators affecting their transcriptional function (reviewed in [46]). To investigate whether histone deacetylases (HDACs) were involved in the COUP-TF II-mediated AR repression, we used the HDAC inhibitors trichostation A (TSA), sodium butylate (NaBut), and nicotinamide (NIC). In PPC-1 cells, the DHT-induced transactivation of AR was inhibited by COUP-TF II coexpression, while it was stimulated by treatment with HDAC inhibitors as previously reported [47], [48]. The relived extent of the repressed AR transactivation by treatment with TSA, NaBut or NIC was not significant compared to the stimulatory effect of relevant HDAC inhibitor itself on AR transactivation (Figure 5B, data not shown). These results suggest that HDACs are not involved in the COUP-TF II-mediated suppression of AR transactivation.

### COUP-TF II Inhibits AR Recruitment to a Target Promoter and Competes with Other Coregulators for the Modulation of AR Transactivation

**Figure 6:**
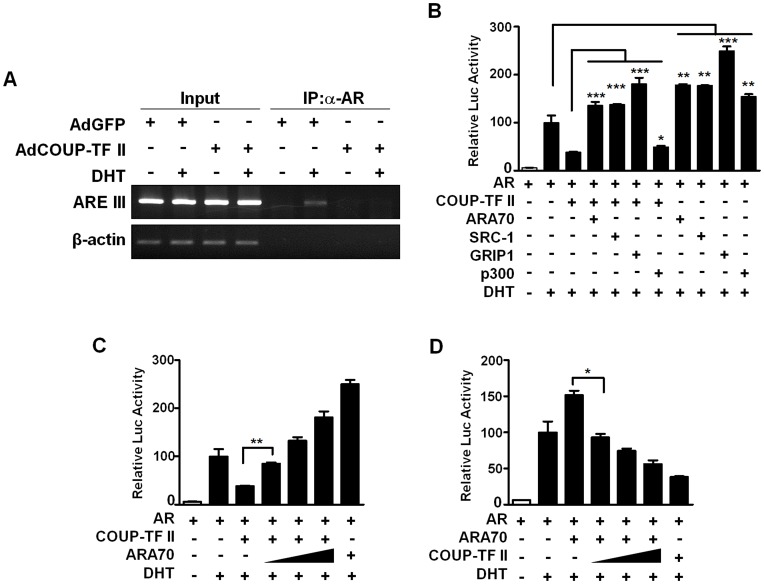
COUP-TF II inhibits AR recruitment to the PSA promoter and competes with AR coactivators to modulate AR transactivation. (A) COUP-TF II inhibits the recruitment of AR to PSA enhancer. LNCaP cells were infected with AdCOUP-TF II or AdGFP. After 24 h of infection, cells were treated with 10 nM DHT or vehicle for 6 h, and then harvested for ChIP assays. Anti-AR antibody (PG-21) was used for immunoprecipitation. Immunoprecipitates were analyzed by PCR using a specific primer pair spanning the AR binding site of the PSA enhancer region. A control PCR for non-specific immunoprecipitation was performed using specific primers to the β-actin coding region. (B) AR coactivators relieve the COUP-TF II-mediated repression of AR transactivation. PPC-1 cells were cotransfected with 50 ng of AR, 250 ng of COUP-TF II and 500 ng of AR coactivator expression plasmids. (C) ARA70 relieves COUP-TF II repression of AR transactivation in a dose-dependent manner. PPC-1 cells were transfected as in “B” with increasing concentration (250, 500, and 1000 ng) of ARA70. (D) COUP-TF II represses ARA70-elevated AR transactivation in a dose-dependent manner. PPC-1 cells were transfected with 50 ng of AR, 250 ng of ARA70 and increasing concentration (250, 500, and 1000 ng) of COUP-TF II expression vector. Cells were treated with or without 3 nM DHT for 24 h. At least three independent experiments were combined and values represent the mean±SEM. *, P<0.05;**, P<0.01; ***, P<0.001.

To explore how COUP-TF II represses AR transactivaiton, we next investigated whether COUP-TF II could affect AR recruitment to the AR target PSA promoter. ChIP assays were performed with LNCaP prostate cancer cells infected with AdGFP or AdCOUP-TF II (Figure 6A). In LNCaP cells infected with AdGFP, the AR was recruited to the ARE-containing enhancer region of the PSA promoter in the presence of DHT, which was, however, strongly reduced by COUP-TF II overexpression in AdCOUP-TF II-infected cells. These results suggest the interference of COUP-TF II with AR binding to the ARE-containing target promoter.

We then examined the possibility that COUP-TF II interferes with the interaction between AR and its coactivators. PPC-1 cells were transfected with plasmids encoding AR and COUP-TF II, and AREx7-tk-luc reporter in the absence or presence of a specific coactivator, and we investigated whether coexpression of a specific coactivator derepresses the COUP-TF II-mediated suppression of AR transactivation. As shown in Figure 6B, ARA70, SRC-1, and GRIP1 relieved the COUP-TF II-induced AR suppression to a certain extent, while p300 did not. Furthermore, ARA70 was able to recover the COUP-TF II-induced AR repression in a dose-dependent manner (Figure 6C), and COUP-TF II was able to repress the ARA70-enhanced AR transactivation in a dose-dependent manner (Figure 6D). Together, these results suggest that COUP-TF II competes with some AR coactivators to modulate AR transactivation.

## Discussion

COUP-TF II acts as a corepressor of nuclear hormone receptors [30], [49], [50]. It has been reported to repress transcription by heterodimerizing with other nuclear hormone receptors, or by interacting with one or several transcriptional coactivator proteins such as HNF-4, HNF-3, and C/EBP [30], [31]. In this study, we demonstrate that COUP-TF II directly interacts with AR and interferes with the N/C terminal interaction of AR, which is probably due to the formation of a heterodimer of COUP-TF II with AR. Therefore, our result suggests that the mechanism of COUP-TF II action for the suppression of nuclear receptors is conserved in some extent for AR.

Corepressors of nuclear receptors are now known to utilize multiple mechanisms to repress the transactivation of nuclear receptors. They include the recruitment of histone deacetylase (HDAC), interference with coactivator interactions, and inhibition of DNA binding activity. Our results showed that COUP-TF II competed with some coactivators such as ARA70, SRC-1, and GRIP1 to modulate AR transactivation. ARA70 and SRC-1 exhibit strong hormone-dependent interaction with the AR LBD through the FXXLF motif within the coactivators, and bridge the AR DBD/LBD complex [51]–[53]. GRIP1 is also capable of binding to both the DBD and LBD of AR, and normally bridges and stabilizes the DBD/LBD complex of AR [54]. Disruption of this AR DBD/LBD/coactivator complex results in the diminution of AR transactivation [52]–[54]. Therefore, the blockage of these coactivators’ binding to AR by COUP-TF II probably disrupts the ternary structure of AR for its transactivation. Recently, we reported that AR transactivation is negatively regulated by HNF-3α via disruption of DBD/LBD/GRIP1 complex [38]. Thus, COUP-TF II probably represses the AR transactivation by a mechanism similar to that for HNF-3α. In contrast, p300, another AR activator, was not able to derepress COUP-TF II-induced suppression of AR transactivation. This is consistent with the fact that p300 activates AR transactivation by inducing the open-structure of chromatin through histone acetylation [47], [55], but not by bridging the DBD/LBD complex of AR. This notion is further supported by our results showing that the HDAC inhibitors TSA, NaBut, and NIC were not able to recover the COUP-TF II-induced repression of AR transactivation.

AR also performs a crucial function in prostate cancer cell proliferation, and thus the levels of COUP-TF II expression may affect prostate cancer growth. Consistent with this prediction, COUP-TF II expression is down-regulated in prostate cancers as compared with the normal prostate in an animal model of prostate cancer, namely Myc-driven transgenic mice [56]. Further, our data show that COUP-TF II expression in human prostate cancer cell lines is strongly down-regulated compared to a normal prostate cell line (Figure 1A). Therefore, COUP-TF II may be associated with the development and progression of prostate cancers, possibly by virtue of its function as an AR corepressor. COUP-TF II has been also reported to inhibit cell growth by blocking cell cycle in MDA-MB-435 cells, ERα-positive and COUP-TF II-negative breast cancer cells [24]. Induction of COUP-TF II in MDA-MB-435 cells resulted in reduced growth, in which cell progression was delayed at G2/M transition phase as a result of the reduction of cdk2 activity. It will be worthwhile to investigate whether cell arrest function of COUP-TF II is also observed in prostate cancer cells and whether the function is related with its inhibitory function of AR transactivation.

In the present study, we have shown that COUP-TF II modulates AR function in prostate cancer cells, affecting androgen-dependent cell proliferation. COUP-TF II prevents the N/C terminal interaction of AR, inhibits AR recruitment to its target promoter, and competes with AR coactivators to modulate AR transactivation. The ability of COUP-TF II to repress AR function and inhibit the growth of prostate cancer cells makes COUP-TF II a new candidate as a therapeutic target for prostate cancers.
